# Video feedback promotes relations between infants and vulnerable first-time mothers: a quasi-experimental study

**DOI:** 10.1186/s12884-017-1568-1

**Published:** 2017-11-15

**Authors:** Ingeborg Hedegaard Kristensen, Marianne Simonsen, Tea Trillingsgaard, Hanne Kronborg

**Affiliations:** 1 Department of Public Health, Section for Nursing, Aarhus University, Høegh-Guldbergs Gade 6A, 8000 Aarhus C, Denmark; 2 Department of Economics and Business Economies, Aarhus University, Fuglesangs Allé 4, 8210 Aarhus V, Denmark; 3 Department of Psychology and Behavioural Sciences, Aarhus University, Bartolins Allé 9, 8000 Aarhus C, Denmark

**Keywords:** Mother-infant interactions, Health visitor, Infant, CARE-index, Marte Meo method

## Abstract

**Background:**

Supporting early mother-infant relationships to ensure infants’ future health has been recommended. The aim of this study was to investigate whether video feedback using the Marte Meo method promotes a healthy early relationship between infants and vulnerable first-time mothers. Video feedback or usual care was delivered by health visitors during home visits in Danish municipalities.

**Methods:**

This quasi-experimental study included pre- and post-tests of 278 vulnerable families. Mothers were allocated to an intervention group (*n* = 69), a comparison group (*n* = 209) and an exactly matched video subsample from the comparison group (*n* = 63). Data consisted of self-reported questionnaires and video recordings of mother-infant interactions. Outcomes were mother-infant dyadic synchrony (CARE-Index), maternal confidence (KPCS), parental stress (PSS), maternal mood (EPDS) and infant socialemotional behaviours (ASQ:SE). The data were analysed using descriptive and linear multiple regression analysis.

**Results:**

The levels of dyadic synchrony in the intervention group had significantly improved (*p* < 0.001) at follow-up with a mean score of 9.51 (95%CI;8.93–10.09) compared with 7.62 (95%CI;7.03–8.21). The intervention group also showed a higher level of maternal sensitivity with a mean score of 9.55 (95%CI;8.96–10.14) compared with 7.83 (95%CI;7.19–8.46) in the matched video subsample (*p* < 0.001). With respect to infant cooperation, similar improvements were found with a mean score of 9.43 (95% CI;8.88–9.99) in the intervention group compared with 7.73 (95%CI;7.13–8.33) in the matched video subsample from the comparison group (*p* < 0.001). Furthermore, mothers in the intervention group reported significantly lower levels of parental stress with a mean score of 32.04 (95%CI;30.13–33.94) compared with 35.29 (95%CI;34.07–36.52) in the comparison group (*p* = 0.03), as well as higher levels of maternal confidence with a mean score of 41.10 (95%CI;40.22–41.98) compared with 40.10 (95%CI;39.65–40.56) in the comparison group (*p* = 0.04). No significant differences were found in EPDS and ASQ:SE.

**Conclusion:**

The findings support the assumption that video feedback using the Marte Meo method early after birth may strengthen the relationship between infants and vulnerable firsttime mothers as well as improve maternal psychosocial functioning. Further research applying random assignment is needed to strengthen these conclusions; further research is also needed to assess any long term effects of the video feedback intervention using the Marte Meo method.

**Trial registration:**

This study was registered on 24 January 2013 in ClinicalTrials.gov with the identifier: NCT01799447.

**Electronic supplementary material:**

The online version of this article (10.1186/s12884-017-1568-1) contains supplementary material, which is available to authorized users.

## Background

The postnatal period has been identified as particularly important for the establishment of early, healthy infant-mother relationships. The nature of this early relationship is associated with infants’ cognitive development [1, 2] as well as their physical [3, 4] and psychosocial health [5–8]. Around 58% of infants in developed countries are securely attached to their mother [9]. In Denmark, 18% of 1½ year old children show symptoms of mental problems [10, 11]. Research on attachment points to the quality of this early relationship as a possible source of such disturbances, and distinguishes between secure and insecure attachment [12] where sensitive parenting may lead to secure attachment and insensitive parenting may lead to insecure attachment. Sensitive parenting is central to infants’ psychosocial functioning [13, 14]. According to Ainsworth et al. [15], the core features of sensitive parenting are acceptance, awareness, responsiveness and cooperation. Following Feldman [5], mothers’ sensitivity can be observed in their ability to engage in positive interactions of synchronous contact with their infants. Thus, sensitive parenting is highly dependent on the mother’s psychosocial functioning [16]. Insensitive parenting may be displayed by mothers who experience abuse, critical illness, domestic conflict and their own or their partner’s mental illness. These mothers have a high risk of developing relationship disorders with their infant in the postnatal period [17–19]. Mothers with low maternal confidence, lack of maternal experience and symptoms of postnatal depression and mothers who have experienced preterm delivery may also be insensitive in the parenting role and have a lower and more hidden risk of developing poorer relationships with their infant [20–24]. First-time mothers may be insecure about responding to the infant’s cues [25, 26]; in particular, mothers who perceive their infant to be crying persistently or who sleep poorly have shown disturbances in establishing early relationships with their infants [27–30]. Maternal insecurity appears to be most pronounced during the first weeks after birth, especially among first-time mothers [31]. A Danish study found that more than half of first-time mothers stated that they had difficulties in soothing their infant during this early period [32]. Approximately 7–14% of new mothers are affected by postnatal depression [16, 33]; these mothers have been found to be less sensitive to their infants [34]. Premature delivery, which is experienced worldwide by 11% of mothers and in developed countries by 6–8% of mothers [35], can also disturb the development of healthy early relationships because the premature infant’s cues may be immature and difficult to read, and the mother may experience psychosocial problems such as crisis, sorrow, stress, uncertainty and helplessness [23, 36]. Thus, there is an extensive body of knowledge about vulnerable mother-infant interactions where mothers and infants may need extra support to build healthy early relationships.

The World Health Organization (WHO) [37] recommends that health professionals support parents in building healthy early relationships. Short standardised parenting programmes focused on new parents’ sensitivity and responsiveness to their infants have a positive impact on mothers’ psychosocial functioning and on mother-infant interactions [38]. In particular, methods using video feedback in home visits in a selected at-risk population of families have shown positive short term effects on infants’ responsiveness to mothers as well as on parental stress and confidence in the parenting role [39–41]. Van Doesum et al. [42] also found evidence of a positive, long term effect. One meta-analysis found stronger positive effects of video feedback when the method was used with low-risk families [43]. Another study found no effects of video feedback in a universal intervention that targeted fathers [44]. The Marte Meo method is a programme that builds on current knowledge of best practice using video feedback to target mothers’ sensitivity and is aimed at developing mothers’ healthy early relationships with their infants [45, 46]. The method is widely used in Scandinavia in interventions that address the quality of the early parent-child relationship. A recent Norwegian randomised, controlled trial targeting families with children from 0 to 2 years with parent-child interaction problems found that the Marte Meo method had positive short term effects on parent-child interactions [47]. A Norwegian qualitative study of mothers with postnatal depression found that the Marte Meo method helped them develop in their parental role [48]. However, no systematic effect evaluation of the Marte Meo in a community setting in the early months after birth has been conducted.

The objectives of the present study were thus to explore whether a standardised video feedback intervention programme using the Marte Meo method in a community setting promoted the development of healthy early relationships between infants and vulnerable first-time mothers.

## Method

### Study design

A quasi-experimental design was used to compare and contrast an intervention group of vulnerable first-time mothers receiving feedback using the Marte Meo method during a 4-month period with a comparison group of mothers receiving standard care. The design included pre-tests at baseline, 2 months postpartum and post-tests 6 months postpartum. Both groups received standard care from health visitors. The intervention group received an additional programme with early intervention using the Marte Meo method designed for this study (Kristensen: Early Intervention with the Marte Meo method - Manual, unpublished).

### Setting

Six municipalities representing 52% of the inhabitants of the invited 17 municipalities in the Central Denmark Region accepted the invitation to participate in the study. The study was conducted from September 2013 to December 2014. In Denmark, all new families receive visits by community health visitors as part of a universal prevention programme that is initiated at discharge from the hospital and continues throughout the first year. Mothers are allocated a health visitor who works within the geographical districts where they reside when the municipal authorities receive birth information from the hospital. Nearly all first-time parents (97–99%) participate in this programme [49]. The programme emphasises health guidance related to infant and parental wellbeing, infant growth, development, nutrition and support in establishing an healthy early parent-infant relationship [49]. Health visitors are registered nurses with 1 year of additional education in infant and child health. Recently, about 20% of the health visitors in the participating municipalities received additional training in giving video feedback using the Marte Meo method.

### Developing the video feedback intervention programme

Elements from the Intervention Mapping approach [50] were used to develop and plan this quasi-experimental study in a community setting. The method addressed the complexity of the intervention study with two study populations: a primary study population of first-time mothers who received the Marte Meo intervention programme or standard care only, and a secondary study population of health visitors who delivered the Marte Meo intervention programme or standard care. The results of the secondary study population of health visitors are described elsewhere [51].

The video feedback intervention programme was based on the Marte Meo method, an approach developed by observation of numerous case studies of parent-infant interactions by Maria Aarts [45]. The Marte Meo method uses video feedback with parents who express concern and need support in relation to their infant and the parenting role [46]. The programme includes video feedback focused on specific guidance aimed at promoting parents’ sensitivity and responses towards their infant. In the present Marte Meo programme, the health visitor video-taped 3–5 min of a parent-infant interaction. This sequence was subsequently analysed by health visitors and prepared for the next home visit which focused on five elements in the parent’s behaviour that were directly observable during the video-taped interaction: 1) waiting, observing and following the infant’s initiative, 2) positively confirming the infant’s initiative, 3) putting own and the infant’s initiatives into words, 4) taking turns with the infant and 5) interacting with positively leadership [46]. During the next home visit, the mother and the health visitor watched the edited replay of the video and subsequently discussed situations where the mother had concrete opportunities to connect with her infant as well as the infant’s response to the mother’s interaction. Afterwards, the mothers were given Marte Meo working points to practise until the next home visit. Similar to the video interaction guiding programme (VIG) [52] and video feedback intervention (VIPP) [41], the programme is intended to promote sensitive parenting. Unlike the VIPP video feedback method with standardised lessons, the number of sessions is flexible since the programme starts with the most pressing concern of the mother and continues until the mother and health visitor agree that the mother’s initial concerns have been resolved. The programme consists of 2–5 additional video feedback sessions in home visits between 2 and 6 months postpartum.

The health visitors in the intervention group were all certified Marte Meo therapists who had recently participated in a 16-h training workshop to brush up on how to assess video interactions according to the Marte Meo method and learn how to use the programme manual developed specifically for the present study (Kristensen: Early Intervention with the Marte Meo method - Manual, unpublished).

### Participating health visitors

All 186 health visitors employed in the six participating municipalities agreed to participate in the study. Among these health visitors, 36 were certified Marte Meo therapists and 90 health visitors had no additional parenting programme education. Together they formed the secondary study population who were to deliver the Marte Meo programme or standard care during the project period. Sixty health visitors were not included in the secondary study population study because they had participated in other standardised parenting programme education programmes than Marte Meo or because they were employed only in school health services.

### Participating mothers

All first-time mothers giving birth during the study period and who lived in a district with a health visitor participating in the study were invited to take part in the study when the health visitor paid her first ordinary home visit and filled in questionnaires at 2 and 6 months postpartum. Mothers were not invited if their Danish language skills were considered insufficient to be able to fill in a Danish language questionnaire. The study population from which the intervention group was recruited comprised mothers living in districts with health visitors certified as Marte Meo therapists. The study population from which the comparison group was recruited comprised mothers living in districts with health visitors without a parenting programme education. The inclusion and exclusion criteria were similar for the intervention and the comparison groups. Included mothers were defined as vulnerable if they met at least one or more of the following three criteria: 1) low parenting confidence score < 40 on the Karitane Parenting Confidence Scale (KPCS), 2) moderate parenting mood score > =8 < 13 on the Edinburgh Postnatal Depression Scale (EPDS) and 3) moderate preterm birth between > = 32 and < 37 gestational weeks. Mothers were excluded if they received treatment in other organisations, had an EPDS score of 13 or more, had given birth prematurely (i.e. < 32 gestational weeks) or were referred to other sectors due to mental illness, abuse including medicine and alcohol, and lack of parental capacity. Also, mothers with infants who suffered from severe disabilities were excluded from the intervention. In the intervention group, invited mothers were screened for vulnerability during the health visitor’s ordinary home visit 7–9 weeks postpartum by asking the mother to fill in the KPCS and the EPDS scales. In the comparison group, mothers were identified as vulnerable based on their reply to the same questions in the baseline questionnaire.

An exactly matched video subsample was ensured by matching one vulnerable mother from the intervention group with one vulnerable mother from the comparison group. The variables used for matching were the following three inclusion criteria: 1) low parenting confidence score < 40 (KPCS), 2) moderate parenting mood score > =8 < 13 (EPDS) and 3) moderate preterm birth between > = 32 and < 37 gestational weeks.

### Data collection

Data consisted of video recordings of mother-infant interactions from the intervention group and matched video subsample from the comparison group, as well as the responses from self-reported questionnaires from the intervention group and the entire comparison group. Three-minute video sequences of mother-infant play were coded using a validated method, the CARE-Index [53], to assess dyadic synchrony, mothers’ sensitive behaviour and infants’ cooperative behaviour in the interactions. In the intervention group, we used the last video recorded during the intervention programme 4–6 months postpartum, and these videos were collected by health visitors in the intervention group. In the comparison group, the exactly matched video subsample from the comparison group of 63 vulnerable mothers was videotaped by a health visitor with a Marte Meo therapist education in the family’s home 5–6 months postpartum.

Questionnaires were used to collect data on maternal socioeconomic group, preparation for parenting, maternal psychosocial functioning and infant birth and social-emotional behaviour. The questionnaires primarily included previously validated and pilot-tested questions, and the questions were reversed in order to ensure their comprehensibility, relevance and acceptability [54]. The baseline questionnaire was sent by e-mail after the researcher had received informed consent from study participants. The e-mail contained a link to a database where mothers could fill in the questionnaire at home 2 months postpartum and a follow-up questionnaire 6 months postpartum. One mother who had no e-mail account received the questionnaire by ordinary mail. Reminders were sent by e-mail and text message after 2 and 3 weeks, respectively.

### Primary outcome measure

The primary outcome measure was mother-infant interaction measured as dyadic synchrony on the CARE-Index [53]. Scores ranged from 0 to 14 with higher scores indicating better dyadic synchrony. In all, 132 video recordings were coded; 69 in the intervention group and 63 in the video subsample of the comparison group. Coding took place from August 2014 to January 2015; codings were performed by the first author and by two health visitors, all certified by Crittenden as research raters in 2014. To ensure inter-rater reliability, a random, blinded sample of 20% of the video recordings was initially coded by the three raters independently. The Cronbach’s alpha coefficient test showed high reliability in rating dyadic synchrony (0.88). To prevent raters’ drift, 10% of the video recordings were drawn randomly from the sample during the coding period, and mother-infant interactions were assessed independently before they were discussed and coded in agreement between the three raters at monthly meetings.

### Secondary observational outcome measures

Maternal sensitivity and infant cooperative behaviour were coded using the CARE-Index [53]. The CARE-Index is used to evalute three aspects of mothers’ behaviour (sensitive, controlling and unresponsive) and four aspects of infants’ behaviour (cooperative, compulsive, difficult and passive). Scores ranged from 0 to 14 concerning the three maternal aspects and four infant aspects giving in total 14 points each with higher scores indicating better and positive results on the measures of maternal sensitivity and infant cooperativeness. Lower scores indicated better and positive results on maternal controlling and unresponsiveness and the infant’s compulsive, difficult and passive behaviour. Cronbach’s alpha coefficients were calculated to report agreement among raters on the triple-coded tapes with regard to the mother’s behaviour (sensitive, controlling and unresponsive) and infant behaviour (cooperative, compulsive, difficult and passive). Cronbach’s alpha was adequate to high (0.70 to 0.88) for mother’s sensitive, controlling and unresponsive behaviour and high for infant’s cooperative, compulsive, difficult and passive behaviour (0.82 to 0.92) [54].

### Secondary self-reported outcome measures

Maternal confidence was assessed using the Karitane Parenting Confidence Scale (KPCS), a 15-item measure with good levels of sensitivity of 81% and a good level of specificity of 88% [55, 56]. Each question was rated on a scale from 0 to 3; values were summed up to a total KPCS, with higher scores indicating higher maternal confidence.

Parental stress was assessed using the Parental Stress Scale (PSS), an 18-item measure with a good level of sensitivity of 81% and a good level of specificity of 83% [57]. Each question was rated on a scale from 1 to 5; values were summed up to a total PSS score, with lower scores indicating lower parental stress.

Maternal mood was assessed using the Edinburgh Postnatal Depression Scale (EPDS), a 10-item measure [58, 59] with an excellent sensitivity level of 96% and an acceptable specificity of 78% in a Norwegian study [60], although a Swedish validation study showed a sensitivity of 96% and an unacceptable specificity of 49% [61]. Each question was rated on a scale from 0 to 3; values were summed up to a total EPDS, with lower scores indicating fewer symptoms of postpartum depression [58].

Infant social-emotional behaviours were assessed at 2 and 6 months using the Ages & Stages Questionnaires (ASQ:SE-2), a 26-item measure with a moderate to good sensitivity of 71–85% and an excellent specificity of 90–98% [62]. The average score was multiplied by the number of unanswered questions and added to the infant’s total score, giving a final total ASQ:SE score, with lower scores indicating fewer problematic emotional behaviours [62]. The self-reported outcome measures are presented in Additional file 1.

### Background and process variables

Background variables collected 2 months postpartum included maternal age, mother born in Denmark, mother’s education and occupation, father’s education and occupation, mother cohabiting with infant’s father, preparing for parenting, severe life events, mother and infant separation postpartum, term birth and infant sex. Process variables collected 6 months postpartum to follow implementation included participation in the intervention, number of home visits from the health visitor, number of home with video feedback, satisfaction with health visitor, satisfaction with video feedback, support from infant’s father and support from peers.

### Study profile

During the study period, 1549 first-time mothers gave birth in the study districts. Of these, 509 mothers were allocated an intervention health visitor; and of these, 316 (74%) mothers completed the baseline questionnaire and 94 (30%) mothers were assessed as vulnerable according to their self-reported responses in the baseline questionnaire. In all, 89 (28%) mothers were screened for vulnerability at home visits by the health visitors. Of these, eight were referred for other treatment and 12 declined to participate in the present study. In all, 69 (22%) mothers received the intervention. At follow-up, 63 mothers in the intervention group filled in the questionnaire and 69 allowed their video recordings to be used as data material.

**Fig. 1:**
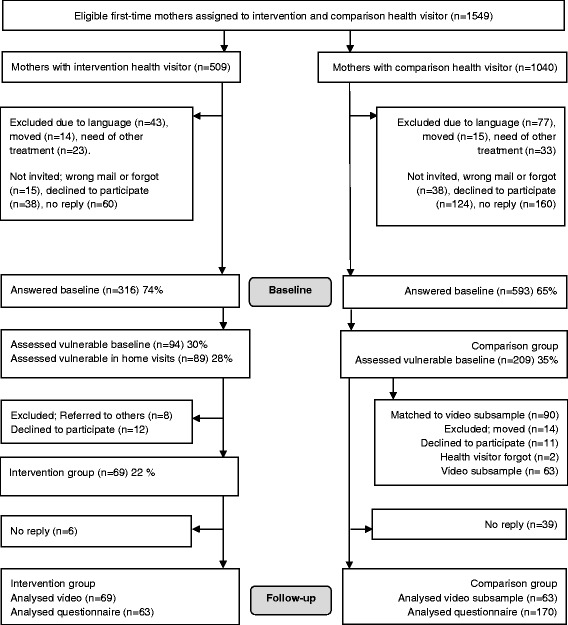
Flow profile of the study population

In all, 1040 mothers were allocated a comparison health visitor, and 593 (65%) mothers completed the baseline questionnaire. Of these, 209 (35%) mothers assessed themselves as being vulnerable according to their self-reported responses in the baseline questionnaire. From the latter group, 90 mothers were matched to be included in the exactly matched video subsample of the comparison group. Of these, 14 were excluded, 11 declined to participate and 2 mothers were not invited. Subsequently, 63 mothers were allocated to the matched video subsample from the comparison group. At follow-up, 170 (81%) vulnerable mothers filled in the questionnaires in the comparison group. A flow profile of the study population is shown in Fig. 1.

### Statistical analysis

Differences in baseline characteristics between groups were analysed. We contrasted the intervention group, first with the entire comparison group and then with the video subsample from the comparison group. Differences in baseline characteristics across intervention and comparison group(s) were tested with unpaired t-tests for continuous, normally distributed variables and with Fisher’s exact test for dichotomous variables.

In the analysis of the observational outcomes, we used the intervention group and the video subsample of the comparison group to test for differences in mean scores. First, we used standard unpaired t-tests for continuous, normally distributed variables to test differences between groups. Second, we used linear multiple regression analysis with the primary outcome, dyadic synchrony, controlling for baseline characteristics (parental education, occupation and stress).

In the analysis of self-reported measures, we used the intervention group and the comparison group. Differences in mean scores across intervention and comparison groups were tested with standard unpaired t-tests. Again, we used linear multiple regression analysis to control each of the self-reported outcome measures at follow-up for differences at baseline including measures of psychosocial functioning at baseline. The inter-rater reliability of the CARE-Index video coding was tested using Cronbach’s alpha. *P*-values below 0.05 were considered significant. STATA version 13.0 was used for all statistical analyses [63].

## Results

### Baseline characteristics

Table 1 provides baseline descriptive statistics for vulnerable first-time mothers allocated to the intervention group and the comparison group. There were no significant differences between the characteristics of the two groups except for mothers in the intervention group where the percentage of mothers with a low educational level was higher (44%, *n* = 28) than in the comparison group (25%, *n* = 50) (*p* < 0.001). Additionally, mothers in the intervention group reported a significantly lower level of parental stress with a mean of 35.15 (SD: 8.31) than mothers in the comparison group with a mean of 37.93 (SD: 7.63) (*p* = 0.02). Additional analysis showed no significant differences between the intervention group and the matched video subsample from the comparison group with regard to mothers’ socioeconomic status, preparation for parenting or mothers’ confidence, mood or having experienced severe life events (results not shown).

**Table 1:** Baseline characteristics of vulnerable first-time mothers and infants comparing intervention and comparison groups

	Intervention group (*n* = 69) Mean (S.D.) *n* (%)	Comparison group (*n* = 209) Mean (S.D.) *n* (%)	*P*-value
Age	30.18 (5.09)	30.45 (3.92)	0.69^c^
Born in Denmark	55 (85%)	186 (91%)	0.18^d^
Cohabiting with infant’s father	60 (92%)	192 (94%)	0.16^d^
Education short	28 (44%)	50 (25%)	**< 0.001** ^d^
Education long	36 (56%)	153 (75%)	
Employed	36 (55%)	124 (63%)	0.26^d^
Unemployed	29 (45%)	72 (37%)	
Partner education short	21 (35%)	59 (31%)	0.99^d^
Partner education long	39 (65%)	131 (69%)	
Partner employed	52 (87%)	148 (80%)	0.42^d^
Partner unemployed	8 (13%)	36 (20%)	
Preparation for parenting			
Antenatal education hours	5.89 (4.25)	7.06 (4.41)	0.08^c^
Previous infant caring: rare/never	36 (56%)	127 (62%)	0.42^d^
Planned pregnancy	58 (89%)	180 (87%)	0.63^d^
Abortion consideration	3 (5%)	7 (3%)	0.63^d^
Boy	25 (38%)	103 (50%)	0.10^d^
Moderate preterm birth	10 (15%)	27 (13%)	0.60^d^
Separated > 2 h postpartum	9 (15%)	20 (10%)	0.53
Social-emotional ASQ:SE^b^	53.36 (30.05)	48.05 (22.83)	0.14^c^
Confidence KPCS^a^	38.59 (4.00)	38.06 (3.33)	0.30^c^
Mood EPDS^b^	6.58 (4.20)	6.96 (3.47)	0.75^c^
Stress PSS^b^	35.15 (8.31)	37.93 (7.63)	**0.02** ^c^
Severe life events^b^	14 (22%)	41 (20%)	0.07^c^

Note: Bold values indicate significant group differences at 5% level. ^a^High score favourable. ^b^Low score favourable. Statistical analysis ^c^Unpaired t-tests. ^d^Fisher’s exact test. Missing values are not included in analysis

### Differences between groups

Table 2 shows the differences between the intervention group and the matched video subsample from the comparison group at 6 months postpartum follow-up. The intervention group showed a significantly higher level of dyadic synchrony with a mean score of 9.51 (95% CI; 8.93–10.09) than the video subsample from the comparison group with a mean score of 7.62 (95% CI;7.03–8.21) (*p* < 0.001). Mothers in the intervention group had a significantly higher level of sensitivity with a mean score of 9.55 (95% CI; 8.96–10.14) than mothers in the video subsample from the comparison group with a mean score of 7.83 (95% CI;7.19–8.46) (*p* < 0.001). Infants in the intervention group showed a significantly higher mean level of being cooperative with a score of 9.43 (95% CI; 8.88–9.99) than infants in the video subsample from the comparison group with a mean score of 7.73 (95% CI;7.13–8.33) (*p* < 0.001). Additional analysis also showed a significant improvement from baseline to follow-up in the intervention group with regard to dyadic synchrony, mothers’ sensitive behaviour and infant’s cooperative behaviour (results not shown).

**Table 2:** Difference in Infant CARE-Index variables between intervention group and the matched video subsample of the comparison group

	Follow-up Intervention group (*n* = 69)	Follow-up Matched video subsample of comparison group (*n* = 63)	*P*-value
CARE-Index variables	Mean (SD) (95% CI)	Mean (SD) (95% CI)	
Dyadic synchrony*	9.51 (2.42) (8.93–10.09)	7.62 (2.34) (7.03–8.21)	**< 0.001** ^**a**^
Mother sensitivity*	9.55 (2.45) (8.96–10.14)	7.83 (2.52) (7.19–8.46)	**< 0.001** ^**a**^
Infant cooperation*	9.43 (2.30) (8.88–9.99)	7.73 (2.38) (7.13–8.33)	**< 0.001** ^**a**^

Note: Bold values indicate significant group differences at 5% level. Missing value = 0 *High score favourable. ^a^unpaired t-tests

Table 3 shows the regression coefficient for dyadic synchrony. In the linear multiple regression analysis, the primary outcome, dyadic synchrony, at follow-up increased with the regression coefficient 1.95 after adjusting the intervention for differences in baseline characteristics of maternal and paternal education, occupation and parental stress at baseline (*p* < 0.001).

**Table 3:** The outcome, dyadic synchrony, at follow-up, controlling for education, occupation and parental stress at baseline

	Dyadic synchrony at 6 months		
Background variables	Coefficient est.	Std. error	*p*-value
Intervention mother	1.95	0.50	**< 0.001**
Mother long education	−0.03	0.58	0.96
Mother employed	−0.16	0.50	0.75
Father long education	0.32	0.52	0.54
Father employed	0.17	0.60	0.78
Parental stress	−0.01	0.03	0.76
Cons	7.80	1.25	**< 0.001**
# observations	109		
Mean of outcome variable	8.73		
R^2^	0.16		

Note: Bold coefficients indicate significance at 5% level. Sample: Intervention and the video subsample of the comparison group. Reference category mothers from the comparison group with short education and no occupation. Missing values are not included in analysis

Table 4 illustrates differences in self-reported questionnaires between the intervention and the comparison group. Significant differences were evident for mothers in the intervention group with a mean level of maternal confidence of 41.10 (95% CI;40.22–41.98) compared with a mean level of 40.10 (95%CI;39.65–40.56) for mothers in the comparison group (*p* = 0.04), and lower mean level for parental stress of 32.04 (95% CI;30.13–33.94) in the intervention group than for mothers in the comparison group at follow-up: 35.29 (95% CI;34.07–36.52) (*p* = 0.03). No significant differences were seen in mothers’ mood and infants’ social-emotional development as measured by the ASQ:SE between the intervention and comparison group. In the linear multiple regression analysis, the self-reported outcomes maternal confidence, mood and parental stress remained robust at follow-up after controlling the intervention for the baseline characteristics maternal education, confidence and mood, and parental stress at baseline (results not shown). In supplemental analyses, infants’ social-emotional development ASQ:SE was significantly lower when the 300 mothers who were allocated a health visitor certified as a Marte Meo therapist were compared with the 533 mothers who were allocated a health visitor without the additional parenting programme education at the 6-month postpartum follow-up (*p* = 0.01) (results not shown).

**Table 4:** Differences between intervention and comparison group at follow-up 6 months postpartum regarding maternal and infant psychosocial functioning

	Follow-up Intervention (*n* = 63)	Follow-up Comparison group (*n* = 170)	
	Mean (SD) (95% CI)	Mean (SD) (95% CI)	*p*-value
Confidence KPCS*	41.10 (3.16) (40.22–41.98)	40.10 (2.96) (39.65–40.56)	**0.04** ^c^
Mood EPDS**	4.8 (3.48) (3.88–5.73)	5.05 (4.02) (4.48–5.72)	0.62^c^
Stress PSS**	32.04 (7.06) (30.13–33.94)	35.29 (7.06) (34.07–36.52)	**0.03** ^c^
Social-emotional development ASQ:SE**	27.76 (16.39) (23.10–31.30)	29.91 (15.97) (27.34–32.23)	0.38^c^

Note: Bold values indicate significant group differences at 5% level. *High score favourable. **Low score favourable. CI: 95% Confidence interval. SD: Standard Deviation. Statistical analysis; ^c^Unpaired t-tests. Missing values are not included in analysis

A process evaluation was conducted to assess whether the programme had been delivered as planned. As intended, mothers in the intervention group received significantly more home visits with a mean of 4.2 [2–6] video feedback sessions; the intervention group received a mean of 8.5 home visits by health visitors in the period from birth to 6 months postpartum, whereas mothers in the comparison group received a mean of 4.6 home visits (*p* < 0.001). When controlling at follow-up for the number of home visits, the primary outcome, dyadic synchrony, remained robust.

## Discussion

### Main results

The present study evaluated a programme that included video feedback with the Marte Meo method versus usual care made available to a selected group of vulnerable first-time mothers and delivered by health visitors in a community setting. The programme was found to increase dyadic synchrony between mother and infant and to be conducive to a more cooperative infant interaction and a more sensitive mother interaction in the intervention group compared with the matched video subsample of the comparison group. The observational outcomes were supported by the self-reported data that showed a significantly higher level of maternal confidence and significantly lower level of parental stress among mothers in the intervention group than among mothers in the comparison group.

### The programme content

The results of the programme may be explained by the combination of video observations and feedback from the health visitors where mothers became more sensitive towards their infants’ interactions when they were guided to read and understand infant cues and their own response to these cues. In a laboratory study, Leavitt [64] discovered that health professionals interpreting infants’ behaviour and cues towards the mother could facilitate maternal sensitivity and responsiveness towards the infant. In observational studies, Ainsworth et al. [15] found that infants’ appearance aroused parents’ feelings and this has later been reported to be a core component in mothers’ understanding of and adequate response to her infant. Our programme included video feedback where health visitors had prepared a selection of video recordings showing where mothers were doing well in their contact with their infants [46]. This delayed video feedback may be superior to health visitors’ home visits because it allows the health visitor to prepare feedback with a selection of positive interactions, and positive feelings may be aroused when mothers observe their own infant’s response. Dialogue may help the mother to see herself from the outside and understand her infant and consequently respond more adequately to the infant’s cues [65]. Our findings showed that trained health visitors using video feedback in home visiting programmes as suggested in the Marte Meo approach may facilitate healthier mother-infant relationships among vulnerable first-time mothers. Further research is needed to determine the impact of video feedback compared with dialogue.

### Results related to other studies

Improved mother-infant interaction is consistent with previous effect studies of interventions that used video feedback in home visiting programmes. Both van Doesum et al. (2008) and Høivik et al. (2015) found that short term positive effects in mother-infant interactions on the Emotional Availability Scales were derived from using video feedback in combination with pedagogical support to target depressed mothers and the Marte Meo method to target families with parent-child (0–2 years) interaction problems, respectively [42, 47]. Hoffenkamp et al. [66] found that an intervention that used the VIG video feedback method was effective as it enhanced parents’ interactions with their preterm infant as measured by video coding by Ravn [66]. Our study thereby supplements earlier findings as it develops a home visiting programme that targets a group of vulnerable families in the early period after birth.

The improvement in mothers’ psychosocial functioning in the intervention group has been identified in other effect studies on home visiting programmes that used different video feedback methods [42, 47, 66]. The higher levels of confidence and lower levels of stress among mothers in the intervention group 6 months postpartum in the present study may be explained by mothers attaining higher degrees of sensitivity in their interactions with their infants as well as the infants’ more cooperative behaviour. Kapp [21] and Jones and Prinz [26] found that if mothers perceive their infants’ behaviour as being less difficult, this bolsters their confidence.

In the present study, infants in the intervention group did not show significantly fewer social-emotional behavioral problems in the short term follow-up measured by the ASQ:SE than infants in the comparison group. However, in the study of the Marte Meo method targeting families with parent-child interaction problems, Høivik et al. [47] found long term effects in ASQ:SE measurements 6 months after the intervention. These findings may suggest that improvements in early infant behaviour may facilitate outcomes in social-emotional behaviour measured by the ASQ:SE in the longer term. A useful follow-up on the present study could thus be to explore whether immediate, positive outcomes in the form of infants becoming more cooperative in their interactions lead to improved social-emotional behaviour. Infants’ social-emotional behaviour may develop as a result of the programme’s promotion of more sensitive parenting. Thus, infants may become more cooperative in their interactions at an early age and show more secure attachments in later life [67].

### Limitations

The quasi-experimental design applied in the present study was the most rigorous method for evaluating the Marte Meo intervention which was already an established practice in some districts in this study. Thus, randomisation of mothers or districts to the intervention or control group was not an option. In order to study differences between groups in a homogeneous study population, we invited first-time mothers because they are characterised by greater insecurity than multiparous mothers and are therefore in need of greater support [31]. The intervention and comparison groups were equal at baseline with regard to maternal confidence, mood and rate of preterm birth, but there were differences in terms of educational level and self-reported stress. To eliminate the possibility of confounding bias, we controlled the outcomes for baseline differences in multiple regression analyses. However, the absence of randomisation is a limitation because other unknown and non-identified confounders may not be balanced across the two groups under comparison. Further research with randomised controlled trials is required to determine whether the improved levels of psychosocial functioning among mothers and infants may be ascribed to participation in the programme. Further research is also needed to assess long term effects of the video feedback intervention using the Marte Meo method.

## Conclusions

This study investigated the effectiveness of a standardised home visiting programme using the Marte Meo method to ensure the establishment of early mother-infant interaction among vulnerable first-time mothers early after birth in a community setting. Although existing results favour the use of the Marte Meo programme in clinical contexts with vulnerable first-time mothers to promote healthy early infant-mother relationships and improve infants’ cooperation and mothers’ sensitivity and psychosocial functioning, more research is needed, such as a randomised controlled study to determine causality in a community setting. Further research is also needed to assess the long term effects of using the Marte Meo method early after birth.

## Additional file

Additional file 1: Questionnaires. English version of the questions used as self-reported outcome measures. (DOCX 19 kb)

## Abbreviations

ASQ:SE: Ages & Stages Questionnaires
EPDS: Edinburgh Postnatal Depression Scale
KPCS: Karitane Parenting Confidence Scale
PSS: Parental Stress Scale
VIG: Video Guiding Interaction
VIPP: Video-feedback Intervention to promote Positive Parenting
