# Supplementary material for: Video feedback promotes relations between infants and vulnerable first-time mothers: a quasi-experimental study
Source: BMC Pregnancy Childbirth. 2017 Nov 15;17:379. doi: 10.1186/s12884-017-1568-1 (PMC5688723; doi:10.1186/s12884-017-1568-1)
Supplement: Additional file 1: — Questionnaires. English version of the questions used as self-reported outcome measures. (DOCX 19 kb) [file 12884_2017_1568_MOESM1_ESM.docx]

Additional file 1

**English version of the questions used as self-reported outcome measures**

*The Karitane Parenting Confidence Scale*
The KPCS 15 items used and scored according to the scoring guideline (57) were the following:

1. I am confident about feeding my baby.
2. I can settle my baby.
3. I am confident about helping my baby to establish a good sleep routine.
4. I know what to do when my baby cries.
5. I understand what my baby is trying to tell me.
6. I can soothe my baby when he/she is distressed.
7. I am confident about playing with my baby.
8. If my baby has a common cold or slight fever, I am confident about handling this.
9. I feel sure that my partner will be there for me when I need support.
10. I am confident that my baby is doing well.
11. I can make decisions about the care of my baby.
12. Being a mother/father is very stressful for me.
13. I feel I am doing a good job as a mother/father.
14. Other people think I am doing a good job as a mother/father.
15. I feel sure that people will be there for me when I need support.

*The Parental Stress Scale*

The PSS 18 items used and scored according to the scoring guideline (59) were the following:

1. I am happy in my role as a parent.
2. There is little or nothing I wouldn't do for my child if it was necessary.
3. Caring for my child sometimes takes more time and energy than I have to give.
4. I sometimes worry whether I am doing enough for my child.
5. I feel close to my child.
6. I enjoy spending time with my child.
7. My child is an important source of affection for me.
8. Having child(ren) gives me a more certain and optimistic view for the future.
9. The major source of stress in my life is my child.
10. Having child(ren) leaves little time and flexibility in my life.
11. Having child(ren) has been a financial burden.
12. It is difficult to balance different responsibilities because of my child.
13. The behaviour of my child is often embarrassing or stressful to me.
14. If I had it to do over again, I might decide not to have a child.
15. I feel overwhelmed by the responsibility of being a parent.
16. Having child(ren) has meant having too few choices and too little control over my life.
17. I am satisfied as a parent.
18. I find my child enjoyable.

*The Edinburgh Postnatal Depression Scale*

The EPDS 10 items used and scored according to the scoring guideline (60) are the following:

1. I have been able to laugh and see the funny side of things.
2. I have looked forward with enjoyment to things.
3. I have blamed myself unnecessarily when things went wrong.
4. I have been anxious or worried for no good reason.
5. I have felt scared or panicky for no very good
6. Things have been getting on top of me.
7. I have been so unhappy that I have had difficulty sleeping.
8. I have felt sad or miserable.
9. I have been so unhappy that I have been crying.
10. The thought of harming myself has occurred to me.

*The Ages & Stages Questionnaires*

The ASQ:SE-2 26 items used and scored according to the scoring guideline (64) are the following:

1. When upset, can your baby calm down within a half hour?

2. Does your baby smile at you and other family members?

3. Does your baby like to be picked up and held?
4. Does your baby stiffen and arch her back when picked up?
5. When talking to your baby, does he look at you and seem to be listening?
6. Does your baby let you know when she is hungry or sick?
7. When awake, does your baby seem to enjoy watching or listening to people?
8. Is your baby able to calm himself down (for example, by sucking on his hand or a pacifier)?
9. Does your baby cry for long periods of time?
10. Is your baby’s body relaxed?
11. Does your baby have trouble sucking from a bottle or breast?
12. Does it take longer than 30 minutes to feed your baby?
13. Do you and your baby enjoy mealtimes together (including breast and bottle feeding)?
14. Does your baby have any eating problems, such as gagging, vomiting, or? (You may write in another problem.)
15. During the day, does your baby stay awake for an hour or longer at one time?
16. Does your baby have trouble falling asleep at naptime or at night?
17. Does your baby sleep at least 10 hours in a 24-hour period?
18. Does your baby get constipated or have diarrhea?
19. Does your baby make sounds and look at you while playing with you?
20. Does your baby make sounds or use gestures to get your attention?
21. When you smile at your baby, does he smile back at you?
22. When you talk or make sounds to your baby, does she make sounds back?
23. Has anyone expressed concerns about your baby’s behavior? If you checked “sometimes” or “most of the time,” please explain:
24. Do you have concerns about your baby’s eating or sleeping behaviors? If so, please explain:
25. Is there anything that worries you about your baby? If so, please explain:
26. What things do you enjoy most about your baby?
